# Financial toxicity, disease acceptance, and health-related quality of life in women with endometriosis: a cross-sectional mediation analysis

**DOI:** 10.3389/fmed.2026.1983927

**Published:** 2026-10-22

**Authors:** Katarzyna Pietrzak, Anna Weronika Szablewska, Marcelina Skrzypek-Czerko, Bartosz Pryba, Monika Tusiewicz, Aleksandra Gaworska-Krzemińska

**Affiliations:** 1 Independent Single-Profile Medical Simulation Unit, Institute of Nursing and Midwifery, Faculty of Health Sciences with the Institute of Maritime and Tropical Medicine, Medical University of Gdańsk, Gdansk, Poland; 2 Department of Obstetric and Gynecological Nursing, Institute of Nursing and Midwifery, Faculty of Health Sciences with the Institute of Maritime and Tropical Medicine, Medical University of Gdańsk, Gdansk, Poland; 3 Division of Neurological and Psychiatric Nursing, Institute of Nursing and Midwifery, Faculty of Health Sciences with the Institute of Maritime and Tropical Medicine, Medical University of Gdańsk, Gdansk, Poland; 4 Division on Nursing Management, Institute of Nursing and Midwifery, Faculty of Health Sciences with the Institute of Maritime and Tropical Medicine, Medical University of Gdańsk, Gdansk, Poland

**Keywords:** disease acceptance, endometriosis, financial toxicity, health-related quality of life, mediation analysis, patient-reported outcomes

## Abstract

**Introduction:**

Endometriosis is a chronic disease associated with substantial impairment in health-related quality of life (HRQoL) and considerable financial burden. This study aimed to examine how patient-reported financial toxicity and disease acceptance are associated with HRQoL in women with endometriosis and to assess the potential mediating role of disease acceptance in this relationship.

**Methods:**

This cross-sectional study included 411 women with endometriosis recruited in Poland through online patient communities, social media channels, endometriosis-related networks, and a collaborating Women's Health Center. Patient-reported financial toxicity, disease acceptance, and HRQoL were assessed using the COST-FACIT, Acceptance of Illness Scale (AIS), and Endometriosis Health Profile-30 (EHP-30), respectively. The potential mediating role of disease acceptance in the association between financial toxicity and HRQoL was examined using regression-based mediation analysis with 5,000 bootstrap samples, adjusting for age, household income, previous surgical treatment, and infertility treatment.

**Results:**

Higher COST-FACIT scores, indicating lower financial toxicity, were associated with greater disease acceptance [B = 0.618, 95% CI (0.552; 0.683), *p* < 0.001] and better HRQoL [B = −0.537, 95% CI (−0.711; −0.362), *p* < 0.001]. Greater disease acceptance was also associated with better HRQoL [B = −0.963, 95% CI (−1.144; −0.784), *p* < 0.001]. Mediation analysis showed a statistically significant indirect association between financial toxicity and HRQoL through disease acceptance [B = −0.595, 95% CI (−0.731; −0.472)], while the direct association remained statistically significant. Older age and previous infertility treatment were independently associated with better HRQoL (lower EHP-30 scores), whereas household income and previous surgical treatment were not.

**Conclusion:**

Patient-reported financial toxicity was associated with both disease acceptance and HRQoL in women with endometriosis. The observed indirect association through disease acceptance is consistent with the possibility that psychological adaptation may represent one component of the relationship between financial toxicity and HRQoL. however, the cross-sectional design does not allow the temporal ordering or causality of these associations to be established. These findings support considering financial well-being alongside psychological factors when evaluating the broader impact of endometriosis on patients’ quality of life.

## 1 Introduction

Endometriosis is a chronic, systemic inflammatory disease whose consequences extend beyond gynecological and reproductive symptoms and affect multiple dimensions of women's health and functioning (1, 2). It is estimated to affect approximately 10% of women of reproductive age worldwide (1, 3). Its clinical presentation is heterogeneous and may include chronic pelvic pain, dysmenorrhea, dyspareunia, fatigue, and infertility. The impact of endometriosis extends across multiple domains of women's lives, including physical functioning, emotional well-being, intimate relationships, social participation, and occupational activity (3–6). Consequently, women with endometriosis commonly report substantially impaired health-related quality of life (HRQoL) and reduced work productivity (4, 5).

Disease-specific patient-reported outcome measures are important for capturing the multidimensional burden of endometriosis from the patient's perspective. The Endometriosis Health Profile-30 (EHP-30) was developed specifically to assess the impact of endometriosis on pain, feelings of control and powerlessness, emotional well-being, social support, and self-image (7). It has since become one of the most frequently used disease-specific instruments for assessing HRQoL in women with endometriosis (8).

In addition to its clinical and psychosocial consequences, endometriosis generates a considerable economic burden. Direct costs arise from repeated medical consultations, diagnostic procedures, pharmacological treatment, surgery, pain management, and infertility-related care. Additional expenses may result from travelling to specialist centres and obtaining services that are unavailable or insufficiently accessible within publicly funded healthcare systems (9–12). Indirect costs include sickness absence, reduced productivity while at work, shorter working hours, changes in employment, and loss of income (4, 9, 13). Previous studies have demonstrated that productivity losses account for a substantial proportion of the overall economic burden of endometriosis (9, 13, 14). Moreover, longer diagnostic delays have been associated with greater healthcare resource utilization and higher costs before diagnosis (10).

These mechanisms may collectively contribute to a cycle in which persistent symptoms, healthcare utilization, and financial strain reinforce one another. Persistent symptoms and delayed diagnosis increase healthcare utilization and disrupt occupational functioning, while reduced work capacity and disease-related expenditures may intensify financial strain. Financial difficulties may, in turn, restrict access to additional diagnostic, therapeutic, rehabilitative, or psychological support and further impair daily functioning. Although such a cycle is clinically plausible, studies examining the economic burden of endometriosis have predominantly focused on healthcare expenditure, productivity loss, and societal costs rather than on the subjective financial experience of individual patients (9, 11, 12, 14, 15).

Financial toxicity has increasingly been recognized as an important patient-reported outcome because it captures the individual experience of financial burden beyond objective healthcare expenditure and productivity losses. The concept was originally developed in oncology, where the Comprehensive Score for Financial Toxicity (COST) was designed and validated to assess patients' perceived financial well-being (16–18). More recently, the Polish COST-FACIT has been psychometrically evaluated in patients with endometriosis. The study demonstrated satisfactory measurement properties and identified several demographic and clinical factors associated with financial well-being, including income, employment, duration of symptoms, distance from healthcare facilities, and healthcare-related expenditure (19). However, that study focused primarily on the psychometric properties and correlates of COST-FACIT and did not examine how patient-reported financial toxicity may be related to disease-specific HRQoL.

The relationship between patient-reported financial toxicity and HRQoL in women with endometriosis is likely to involve psychological adaptation to chronic illness. Disease acceptance reflects the extent to which a person adapts to illness-related limitations and incorporates the disease into everyday life without excessive feelings of dependence, loss of control, or diminished self-worth. Previous studies have shown that greater acceptance of endometriosis is associated with better quality of life (20–22). Pain, maladaptive pain-coping strategies, infertility, income, and selected disease-related characteristics have also been examined as potential determinants of illness acceptance (23). Disease acceptance was considered particularly relevant because it reflects a relatively stable process of psychological adaptation to chronic illness, integrating patients' responses to illness-related limitations and everyday functioning rather than symptom severity alone. In addition, recent research has confirmed that psychological resources and diagnostic and socioeconomic factors are relevant to the quality of life of women with endometriosis (20, 24). Therefore, the unresolved question is not whether disease acceptance or socioeconomic circumstances are associated with (9, 13–15) HRQoL, but whether disease acceptance constitutes a potential psychological pathway linking patient-reported financial toxicity with disease-specific quality of life.

Financial toxicity may be relevant to psychological adaptation because it reflects not only the economic consequences of illness but also the subjective experience of financial strain. In women with endometriosis, treatment-related expenses, repeated healthcare utilization, and reduced work capacity may contribute to financial strain which may increase the perceived burden of living with the disease and challenge adaptation to its persistent limitations (9, 13–15). From this perspective, greater financial strain may be associated with lower disease acceptance, reflecting greater difficulty in psychologically adapting to the consequences of chronic illness (22).

Disease acceptance, in turn, reflects the extent to which individuals adapt to illness-related limitations and integrate them into everyday life. Greater acceptance has been associated with better quality of life in women with endometriosis, suggesting that psychological adaptation may represent one process through which contextual and illness-related burdens are associated with HRQoL (20, 24). However, disease acceptance represents only one aspect of psychological adaptation in endometriosis. Recent studies indicate that other potentially modifiable cognitive and adaptive processes, including pain catastrophizing and body functionality appreciation, may contribute to specific psychological and functional outcomes in women with endometriosis, highlighting the complex interplay between symptoms, psychological responses, and well-being (25, 26).

The hypothesised relationships can be conceptually situated within the revised Wilson-Cleary model of HRQoL, which links biological and clinical factors, symptom status, individual and environmental characteristics, functional status, health perceptions, and overall quality of life (27, 28). Within this framework, financial toxicity may be regarded as a contextual stressor related to the economic consequences of illness, whereas disease acceptance may represent a psychological adaptation process associated with patients' perceptions of and responses to their condition. Accordingly, psychological adaptation may represent one potential psychosocial factor involved in the association between contextual financial burden and patients' functioning, health perceptions, and ultimately HRQoL. Although these constructs have previously been examined separately or in pairwise relationships, their interrelationships have not, to our knowledge, been evaluated within an integrated mediation model in women with endometriosis.

While our previous work focused on the translation, psychometric validation, and basic correlates of the Polish COST-FACIT, the present study extends this work by examining the clinical and psychosocial relevance of patient-reported financial toxicity in endometriosis, specifically in relation to disease acceptance and disease-specific HRQoL.

Therefore, the aim of this study was to examine the associations between patient-reported financial toxicity, disease acceptance, and disease-specific HRQoL in women with endometriosis, assess the potential mediating role of disease acceptance in the association between financial toxicity and HRQoL. We hypothesised that greater patient-reported financial toxicity would be associated with poorer HRQoL and that disease acceptance would statistically mediate this association after accounting for selected sociodemographic and clinical factors.

## 2 Material and methods

### 2.1 Study design and participants

This cross-sectional study was conducted during the fourth quarter of 2025 using the Computer-Assisted Web Interview (CAWI) method. The present study is a secondary analysis of data collected as part of the COST-FACIT-PL validation project previously reported by Pietrzak et al. (19). While the previous study focused on psychometric validation of the COST-FACIT-PL, the present analysis addresses the associations between financial toxicity, disease acceptance, and HRQoL. Data were collected through an online survey administered via the LimeSurvey platform. Participants were recruited through multiple Polish online channels, including publicly accessible social media channels, closed online patient communities, endometriosis-related advocacy networks, and a collaborating Women's Health Center. Membership in closed patient communities required administrator approval. The survey was distributed via an open link without individual token restrictions. No identifying personal information was required to participate in the main survey. Participants could voluntarily provide an email address for the test–retest component of the broader validation study. Email addresses were stored separately from survey responses to preserve confidentiality. The provided email addresses were also screened for repeated entries as part of data quality assessment; no duplicate email addresses were identified. Because provision of an email address was optional, duplicate participation among respondents who did not provide an email address could not be completely excluded. The survey remained anonymous for participants who did not provide an email address for the test–retest procedure.

Women aged 18 years or older were eligible to participate if they provided electronic informed consent and self-reported a diagnosis of endometriosis previously established by a healthcare professional. Consistent with contemporary international clinical recommendations, surgical or histopathological confirmation was not required. Eligible diagnoses included those established based on surgical findings, with or without histopathological confirmation, or on clinical and/or imaging findings considered consistent with endometriosis (2). Participants reporting self-diagnosis without prior medical evaluation or whose responses provided insufficient information to establish eligibility were excluded. The diagnosis was self-reported and was not independently verified against medical records. Additional information on endometriosis-related clinical history, including age at diagnosis, diagnostic methods, and previous treatment, was collected to characterize the participants and assess the internal consistency of responses.

No formal *a priori* sample size calculation was performed for the present study because it represents a secondary analysis of an existing dataset. The analytical sample was determined by the number of eligible participants who completed the survey during the predefined recruitment period and met the data quality criteria. A total of 983 women initiated the survey, of whom 504 completed all questionnaires. During data quality assessment, 28 records were excluded due to implausible or internally inconsistent responses, resulting in a dataset of 476 participants. Prior to the regression-based mediation analysis, model diagnostics were performed to identify influential observations using Cook's distance and Mahalanobis distance. Cases meeting either diagnostic criterion – a Mahalanobis distance greater than 2.0 or a Cook's distance exceeding 4/n – were considered influential and excluded from the primary analysis. Based on these diagnostic criteria, 65 observations were excluded from the analysis, resulting in a final analytical sample of 411 participants. A sensitivity analysis was conducted by re-estimating the mediation model in the full post-quality-control sample (*N* = 476), with influential observations retained, and comparing the results with those of the primary analysis (*N* = 411). The direction and statistical significance of the associations among financial toxicity, disease acceptance, and HRQoL were consistent across both analyses, supporting the robustness of the main findings. Some differences were observed for individual covariates; however, these did not alter the overall pattern of associations among the primary study variables. Detailed results of the sensitivity analysis are presented in Supplementary Table S1. Participant flow and reasons for exclusion are presented in Figure 1.

**Figure 1:**
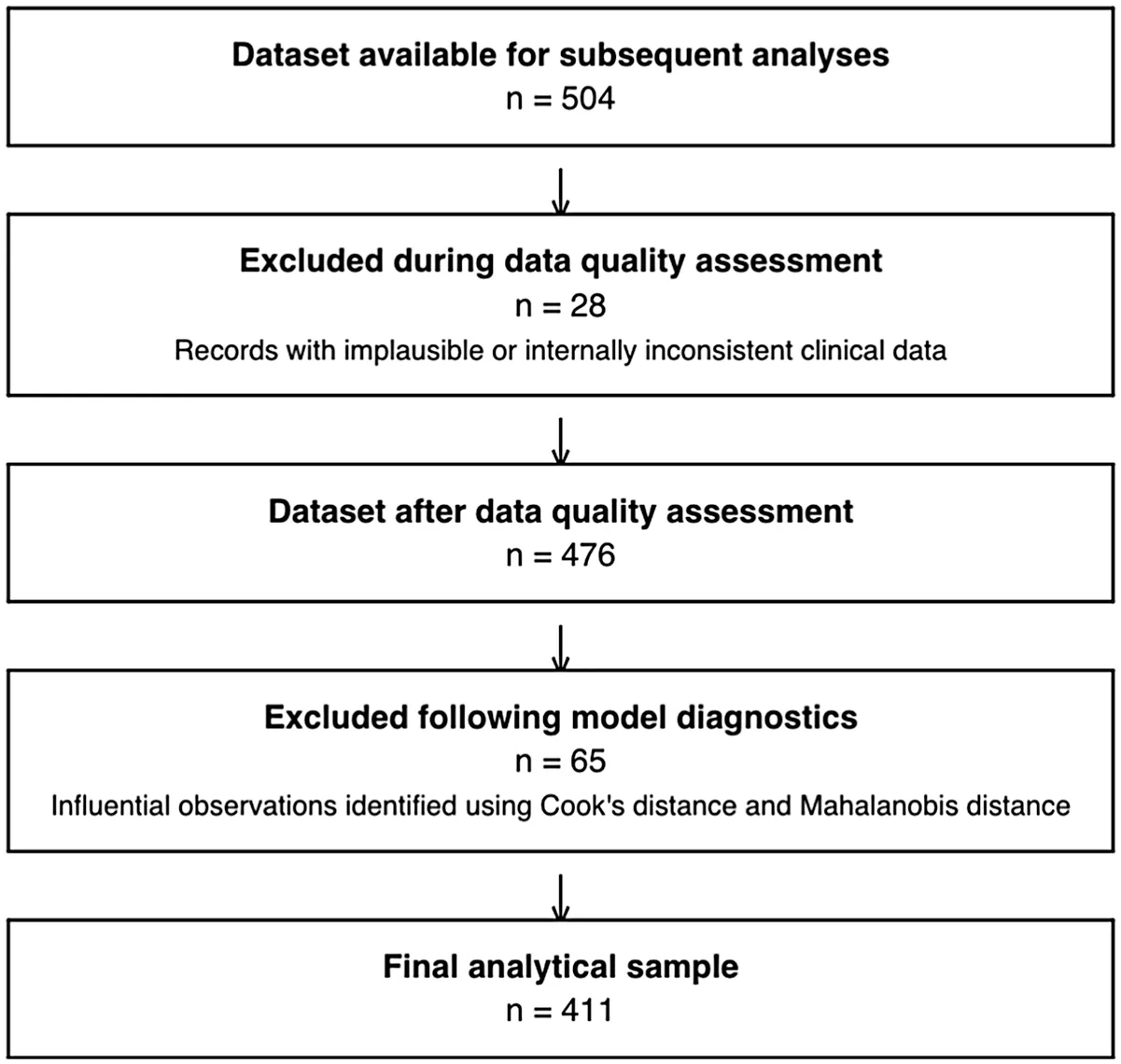
Flow diagram of participant inclusion and exclusions for the analytical sample.

This manuscript was prepared in accordance with the Strengthening the Reporting of Observational Studies in Epidemiology (STROBE) guidelines for cross-sectional studies (29). The completed STROBE checklist is provided as Supplementary Table S2.

### 2.2 Ethical considerations

The study was conducted in accordance with the principles of the Declaration of Helsinki and was approved by the Independent Bioethics Committee for Scientific Research at the Medical University of Gdańsk (approval no. KB/517-460/2025). Electronic informed consent was obtained from all participants before completion of the survey.

### 2.3 Questionnaire

#### 2.3.1 Sociodemographic clinical, and disease-related characteristics

The questionnaire was developed as part of a broader research project and collected a wide range of sociodemographic, clinical, disease-related, and healthcare-related information. For the purposes of the present study, a subset of variables relevant to the study objectives was used to characterize the study sample and conduct the planned analyses. The characteristics used to describe the study sample included age, education, place of residence, marital status, number of children, employment status, household income, diagnostic delay, previous surgical treatment, previous infertility treatment, and self-reported diagnosis- and treatment-related costs. Of these variables, age, household income, previous surgical treatment, and previous infertility treatment were included as covariates in the mediation analysis.

#### 2.3.2 Patient-reported financial toxicity

Patient-reported financial toxicity was assessed using the Polish version of the Comprehensive Score for Financial Toxicity (COST-FACIT), a patient-reported outcome measure developed to assess patient-reported financial toxicity associated with disease and its treatment. The instrument consists of 11 scored items and one summary item (FT12), which is not included in the total score calculation. Responses are rated on a five-point Likert scale, and total scores range from 0 to 44, with higher scores indicating better perceived financial well-being and lower financial toxicity (16, 17). The Polish version of the COST-FACIT (COST-FACIT-PL), adapted and validated by Pietrzak et al. (18), demonstrated satisfactory reliability and validity in women with endometriosis, with a Cronbach's alpha of 0.92 (19).

#### 2.3.3 Health-related quality of life

Health-related quality of life (HRQoL) was assessed using the Endometriosis Health Profile-30 (EHP-30), a disease-specific patient-reported outcome measure developed to assess the impact of endometriosis on women's HRQoL (7). The EHP-30 consists of a 30-item core questionnaire covering five domains: pain, control and powerlessness, emotional well-being, social support, and self-image. Responses are rated on a five-point Likert scale ranging from 0 (“never”) to 4 (“always”). The EHP-30 Summary Index was used as the primary HRQoL outcome measure, which ranges from 0 to 100, with higher scores indicating poorer HRQoL. In the original validation study, the EHP-30 core domains demonstrated high internal consistency, with Cronbach's alpha coefficients ranging from 0.83 to 0.93 (7). The official Polish version of the EHP-30, obtained from the University of Oxford with the required authorization, was used in this study, and scoring was performed according to the official scoring instructions.

#### 2.3.4 Disease acceptance

Disease acceptance was assessed using the Acceptance of Illness Scale (AIS), a patient-reported outcome measure developed to evaluate the extent to which individuals adapt to the limitations and consequences of living with a chronic disease. The instrument consists of eight items rated on a five-point Likert scale, yielding a total score ranging from 8 to 40. Higher scores indicate greater disease acceptance. The Polish version of the AIS was adapted and validated by Juczyński and demonstrated satisfactory internal consistency, with a Cronbach's alpha of 0.82 (21, 22).

### 2.4 Potential sources of bias

Potential sources of bias were considered during study design, recruitment, and data preparation. To reduce reliance on a single recruitment source, the survey was disseminated through multiple channels, including publicly accessible social media platforms, closed online patient communities, endometriosis-related networks, and a collaborating Women's Health Center. Membership in closed patient communities required administrator approval. Nevertheless, the non-probability recruitment strategy may have resulted in self-selection of women who were more engaged with endometriosis-related content or more willing to report their experiences. Eligibility was based on a self-reported diagnosis of endometriosis made by a healthcare professional. Although the diagnosis could not be independently verified against medical records, detailed information on diagnostic history, diagnostic methods, and previous treatment was collected to assess the internal consistency of participants' responses. As part of data quality assessment, records containing implausible or internally inconsistent responses were identified and excluded prior to the statistical analysis. Potential duplicate participation was assessed by screening voluntarily provided email addresses for repeated entries; no duplicate email addresses were identified. However, because provision of an email address was optional and the survey was distributed via an open link without individual token restrictions, duplicate participation among respondents who did not provide an email address could not be completely excluded.

### 2.5 Statistical analysis

Statistical analyses were performed using IBM SPSS Statistics version 31.0 (IBM Corp., Armonk, NY, USA). Descriptive statistics were used to summarize participants' sociodemographic and clinical characteristics. Continuous variables are presented as means and standard deviations (SD), whereas categorical variables are presented as frequencies and percentages. Missing data were handled using complete-case analysis; observations with missing values for variables required for a given analysis were excluded, and no data imputation was performed.

The hypothesized mediating role of disease acceptance in the association between patient-reported financial toxicity and health-related quality of life (HRQoL) was examined using regression-based mediation analysis implemented with the PROCESS macro for SPSS (30). Given that all variables were assessed concurrently, the mediation analysis was intended to statistically decompose the observed cross-sectional associations and not to establish temporal or causal relationships. Bootstrap resampling with 5,000 samples was used to estimate standard errors (SEs) and 95% confidence intervals (CIs) for the regression coefficients and indirect effect. The indirect effect was considered statistically significant when the corresponding 95% bootstrap CI did not include zero.

Potential confounding variables were selected based on substantive considerations and evidence from previous literature regarding factors potentially associated with financial toxicity, disease acceptance, and HRQoL in women with endometriosis. The mediation analysis was adjusted for age, household income, previous surgical treatment, and infertility treatment. These variables were selected because they represent key sociodemographic, economic, and treatment-related characteristics with plausible associations with the primary study variables and were available in the existing dataset. Age was treated as a background sociodemographic characteristic, while previous surgical treatment and infertility treatment reflected treatment history preceding survey completion. Household income per capita reflected participants' socioeconomic circumstances at the time of assessment. Given the cross-sectional design, temporal ordering between household income and the concurrently assessed exposure (financial toxicity), mediator (disease acceptance), and outcome (HRQoL) cannot be established.

In the regression models, age was entered as a continuous variable, whereas household income per capita, originally collected in four predefined categories, was dichotomized into ≤5,000 PLN and >5,000 PLN per month. The cut-off was selected to distinguish participants in the lower income range within the Polish socioeconomic context, rather than to model a graded association across income levels. Because income was collected in predefined categories rather than as a continuous measure, more detailed variation within each income category could not be assessed.

Prior to the analyses, the assumptions underlying linear regression were assessed, including linearity, normality of residuals, homoscedasticity, and the absence of multicollinearity. Visual inspection of scatterplots indicated no apparent violations of the linearity assumption. Residual normality was evaluated using the Kolmogorov–Smirnov test, whereas homoscedasticity and multicollinearity were assessed using the Breusch-Pagan test and variance inflation factors (VIFs), respectively. The Kolmogorov–Smirnov and Breusch-Pagan tests were non-significant for both regression models, and VIF values ranged from 1.13 to 2.14, indicating no evidence of problematic multicollinearity. Detailed diagnostic results for all regression models are provided in Supplementary Table S3. Statistical significance was set at *p* < 0.05.

## 3 Results

### 3.1 Participant characteristics

A total of 411 women with endometriosis were included in the analysis. Detailed sociodemographic and clinical characteristics of the study sample are presented in Table 1. There were no missing data for the variables included in the final analytical sample.

**Table 1:** Sociodemographic and clinical characteristics of the study participants.

Variables			*n*	%
Sociodemographic	Age	18–25 years	68	16.55
		26–35 years	204	49.64
		36–45 years	122	29.68
		Over 45 years	17	4.14
	Education level	Primary	4	0.97
		Vocational	10	2.43
		Secondary	101	24.57
		Higher	296	72.02
	Place of residence	Village	75	18.25
		Small urban area <150 000	124	30.17
		Medium-sized urban area 150 000–500 000	83	20.19
		Large urban area >500 000	129	31.39
	Marital status	Single	90	21.90
		Informal partnership	100	24.33
		Married	206	50.12
		Divorced/Widowed	15	3.65
	Children	No	280	68.13
		Only one	74	18.00
		More than one	57	13.87
	Employment status	Unemployed	39	9.49
		Temporary	24	5.84
		Maternity leave	16	3.89
		Sick leave	21	5.11
		Permanent	311	75.67
	Income [PLN]	Under 3,000	66	16.06
		3,000–5,000	110	26.76
		5,001–8,000	131	31.87
		>8,001	104	25.30
Clinical Factors	Surgical treatment	No	226	54.99
		Yes	185	45.01
	Infertility treatment	No	309	75.18
		Yes	102	24.82
	Diagnostic delay	0–5 years	128	31.14
		6–10 years	127	30.90
		11–20 years	141	34.31
		>20 years	15	3.65
	Diagnosis costs [PLN]	0–2,000	110	26.76
		2,001–5,000	129	31.39
		5,001–10 000	86	20.92
		10 000–50 000	78	18.98
		>50 001	8	1.95
	Treatment costs [PLN]	0–2,000	105	25.55
		2,001–5,000	91	22.14
		5,001–10 000	80	19.46
		10 000–50 000	99	24.09
		>50 001	36	8.76
	Total costs [PLN]	0–5,000	89	21.65
		5,001–15 000	152	36.98
		15 001–50 000	96	23,36
		50 001–100 000	54	13.14
		>100 001	20	4.87

### 3.2 Regression-based mediation analysis

The regression model predicting disease acceptance was statistically significant and explained 50.2% of the variance in Acceptance of Illness Scale (AIS) scores [R^2^ = 0.502, adjusted R^2^ = 0.496, *F*(5, 405) = 81.632, *p* < 0.001]. Patient-reported financial toxicity significantly and positively associated with disease acceptance [*B* = 0.618, 95% CI (0.552; 0.683), *p* < 0.001]. None of the covariates, including household income, age, previous surgical treatment, or infertility treatment, were significantly associated with disease acceptance. Detailed regression coefficients are presented in Table 2.

**Table 2:** Regression-based mediation analysis examining the association between patient-reported financial toxicity and health-related quality of life through disease acceptance.

Antecedent	Consequent											
	Acceptance of illness [AIS]						Quality of life [EHP-30]					
	*B*	*SE*	*95% CI*		*t*	*p*	*B*	*SE*	*95% CI*		*t*	*p*
			*LL*	*UL*					*LL*	*UL*		
COST-FACIT score	0.618	0.034	0.552	0.683	17.376	<0.001	−0.537	0.087	−0.711	−0.362	−6.109	<0.001
Acceptance of illness [AIS]	––	––	––	––	––	––	−0.963	0.091	−1.144	−0.784	−10.361	<0.001
Income ≤5 000 → > 5 000	0.777	0.668	−0.582	2.060	1.225	0.221	0.957	1.148	−1.234	3.278	0.805	0.421
Age	0.083	0.042	0.002	0.166	1.868	0.063	−0.229	0.079	−0.380	−0.072	−2.744	0.006
Surgical treatment No → Yes	−1.074	0.638	−2.300	0.178	−1.658	0.098	1.074	1.165	−1.233	3.303	0.884	0.377
Infertility treatment No → Yes	−0.033	0.637	−1.272	1.204	−0.047	0.963	−3.935	1.256	−6.305	−1.427	−2.998	0.003
Constant	9.558	1.356	6.963	12.283	6.624	<0.001	101.254	2.703	96.093	106.649	35.633	<0.001
Model fit	$R^{2}=\;0.502,R_{\mathrm{adj}}^{2}=\;0.496,$ *F*_(5, 405)_ = 81.632, *p* < 0.001						$R^{2}=\;0.550,R_{\mathrm{adj}}^{2}=\;0.543,$ *F*_(6, 404)_ = 82.158, *p* < 0.001					

AIS, Acceptance of Illness Scale; COST-FACIT, Comprehensive Score for Financial Toxicity; EHP−30– Endometriosis Health Profile; *B*, unstandardized regression coefficient; *SE*, bootstrap standard error; *CI*, bootstrap confidence interval; *LL*, lower limit; *UL*, upper limit; *t*, *t*-statistic; *p*, *p*-value; *R*^2^, coefficient of determination; adjusted *R*^2^, adjusted coefficient of determination; *F*, *F*-statistic. Standard errors and 95% confidence intervals were estimated using 5,000 bootstrap samples.

The regression model with health-related quality of life (HRQoL) as the outcome was statistically significant and explained 54.3% of the variance in HRQoL (R^2^ = 0.550, adjusted R^2^ = 0.543). Household income and previous surgical treatment were not related to HRQoL in the adjusted model. Older age and a history of infertility treatment were associated with lower EHP-30 scores, indicating better HRQoL. The strongest associations with HRQoL were observed for patient-reported financial toxicity and disease acceptance. To facilitate interpretation of the magnitude of these associations, each 1-point increase in COST-FACIT score (indicating better financial well-being and lower financial toxicity) was associated with an estimated 0.62-point increase in AIS score and a 0.54-point decrease in EHP-30 score, after adjustment for the included covariates. Each 1-point increase in AIS score was, in turn, associated with an estimated 0.96-point decrease in EHP-30 score, indicating better HRQoL. The main results of the mediation analysis are presented in Figure 2. Including disease acceptance as a mediator significantly increased the explanatory power of the model [*ΔR*^2^ = 0.120, *ΔF*_(1,404)_ = 107.349, *p* < 0.001].

**Figure 2:**
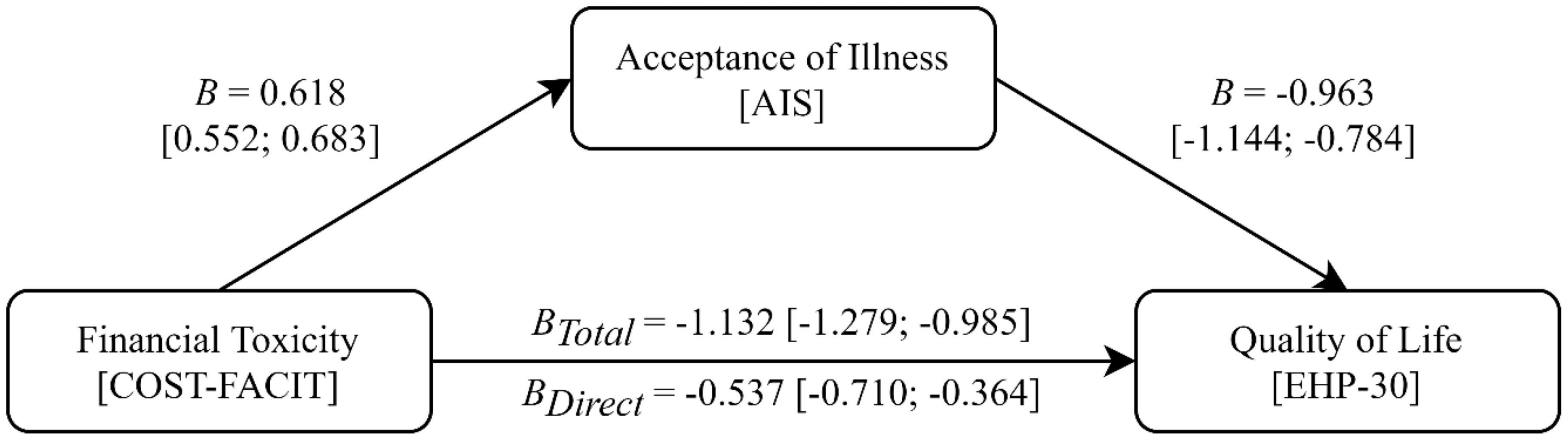
Path diagram of the mediation model examining illness acceptance as a mediator between financial toxicity and quality of life. The figure presents unstandardized regression coefficients with 95% confidence intervals shown in brackets. All paths were estimated while controlling for income, age, surgical treatment, and infertility treatment. Arrows represent the direction specified in the statistical model and should not be interpreted as evidence of temporal or causal relationships AIS – Acceptance of Illness Scale, COST-FACIT – Comprehensive Score for Financial Toxicity, EHP – Endometriosis Health Profile.

The indirect association between patient-reported financial toxicity and health-related quality of life (HRQoL) through disease acceptance was statistically significant [*B* = −0.595, 95% CI (−0.731; −0.472)]. The findings were consistent with partial statistical mediation, as both the direct association between patient-reported financial toxicity and HRQoL and the indirect association through disease acceptance were statistically significant. Lower financial toxicity (higher COST-FACIT scores) was associated with higher levels of disease acceptance, whereas greater disease acceptance was associated with better HRQoL.

## 4 Discussion

This study examined the associations between patient-reported financial toxicity, disease acceptance, and health-related quality of life (HRQoL) in women with endometriosis, with particular emphasis on the potential mediating role of disease acceptance. Higher COST-FACIT scores, indicating better financial well-being and lower financial toxicity, were associated with better HRQoL and greater disease acceptance, while greater disease acceptance was associated with better HRQoL. Importantly, the indirect association between financial toxicity and HRQoL through disease acceptance was statistically significant. The direct association between financial toxicity and HRQoL also remained statistically significant, with the findings therefore being consistent with partial statistical mediation. The regression coefficients provide an indication of the magnitude of the observed associations in the original scale units, but the clinical significance of these differences cannot be established from the present analysis. Taken together, these findings suggest that disease acceptance may represent one psychosocial factor involved in the association between patient-reported financial toxicity and HRQoL in women with endometriosis.

Although the economic burden of endometriosis has been increasingly recognized, considerably less attention has been paid to how patients' subjective experience of financial strain relates to their quality of life. Financial toxicity has been highlighted as an emerging patient-level concern in endometriosis, arising not only from out-of-pocket healthcare expenditures but also from treatment-related costs, reduced work capacity, and financial barriers to accessing care (31). The Polish COST-FACIT has been validated specifically in women with endometriosis, supporting the reliable assessment of financial toxicity as a patient-reported construct in this population (19). Building on this evidence, the present findings extend the relevance of financial toxicity beyond its sociodemographic and clinical correlates by demonstrating its independent association with HRQoL.

The Polish healthcare context may also be relevant when interpreting the observed financial burden. Although Poland has a predominantly publicly financed healthcare system, out-of-pocket payments remain an important component of healthcare expenditure, and access to publicly funded specialist care may be constrained by long waiting times, which may encourage patients who can afford it to seek care in the private sector (32). These system-level constraints may be particularly relevant in endometriosis. Recent Polish evidence indicates that limited access to specialized diagnostic expertise within the public healthcare system may lead patients to seek diagnostic services privately, thereby generating additional out-of-pocket costs (33). Private diagnostic imaging for endometriosis, including transvaginal ultrasound and magnetic resonance imaging, has also been shown to involve substantially higher patient costs than corresponding services in the public sector (33). These characteristics of the Polish healthcare system provide important context for interpreting patient-reported financial toxicity in the present study.

The observed association between patient-reported financial toxicity and HRQoL may reflect the multidimensional nature of financial toxicity, which extends beyond objective healthcare expenditure and encompasses material, psychological, and behavioral consequences of financial hardship (34, 35). In women with endometriosis, perceived financial strain may therefore be related to poorer HRQoL not only through disease-related expenses, but also through financial insecurity, concerns about meeting everyday needs, psychological distress, and financial coping behaviors. This interpretation is supported by evidence from other clinical populations Evidence from oncology, where financial toxicity has been most extensively studied, has consistently demonstrated an association between greater financial toxicity and poorer HRQoL across different patient populations and dimensions of quality of life (36–41). These findings provide a useful context for interpreting our results because the concept of financial toxicity and much of the evidence involving the COST-FACIT have been developed primarily in oncology. However, extrapolation to women with endometriosis requires caution. Unlike many oncological conditions, endometriosis is a chronic, non-malignant disease characterized by a distinct disease trajectory, prognosis, and pattern of healthcare utilization and treatment burden. Therefore, evidence from oncology should be regarded as supportive rather than directly comparable. Nevertheless, the consistency of the association observed across different clinical populations supports our finding that subjective financial burden is closely associated with HRQoL in women with endometriosis, while the mechanisms underlying this relationship in endometriosis require further investigation.

In the present analysis, higher COST-FACIT scores, reflecting better financial well-being and lower financial toxicity, were strongly associated with greater disease acceptance. This finding suggests that psychological adaptation to endometriosis may be related to the subjective experience of financial strain. Direct evidence regarding the relationship between patient-reported financial toxicity and illness acceptance remains scarce; however, a similar association has been observed in women with breast cancer, in whom greater financial difficulties were associated with lower illness acceptance (42). Studies examining socioeconomic indicators alone have yielded a more complex picture. In women with endometriosis, Andysz and Merecz-Kot found that income was not an independent predictor of illness acceptance, consistent with the absence of an independent association between household income and AIS in our study (23). Taken together, this evidence indicates that disease acceptance may be more closely associated with perceived financial strain than with income alone.

Disease acceptance was also independently associated with HRQoL in the present study, with greater acceptance corresponding to better HRQoL. This association may reflect the role of illness acceptance in psychological adaptation to living with a chronic and potentially disruptive condition. Greater acceptance may be accompanied by a better ability to accommodate disease-related limitations within everyday life, whereas difficulties in adapting to persistent symptoms and their consequences may contribute to poorer physical, psychological, and social functioning. This interpretation is supported by previous evidence in women with endometriosis. Bień et al. found that greater illness acceptance was associated with better quality of life across multiple domains, including physical and psychological health and social relationships (24). More recently, Khan et al., using the AIS and WHOQOL-BREF in women with physician-diagnosed endometriosis, also identified illness acceptance as a significant predictor of quality of life and emphasized its potential role in helping women navigate the challenges associated with the disease (43). Similarly, Andysz and Merecz-Kot found that lower pain frequency and less pain catastrophizing were associated with greater illness acceptance, suggesting that the physical and psychological burden of persistent symptoms may hinder adaptation to endometriosis (23). Evidence from other chronic disease populations further supports a positive relationship between illness acceptance and different dimensions of HRQoL (44–46). The association observed in our study may therefore reflect a broader adaptive process in which the way women adjust to the limitations and consequences of endometriosis is closely related to their perceived quality of life.

Illness acceptance should not be interpreted as the resolution of disease or the absence of symptoms. Rather, it reflects psychological adaptation to the limitations and changes imposed by a chronic condition and the integration of these consequences into everyday life (23). This perspective may be particularly relevant in endometriosis, a chronic condition characterized by persistent or recurrent symptoms, long-term treatment, and limitations affecting everyday, occupational, social, and reproductive functioning (23, 47). In this context, greater disease acceptance may facilitate adaptation to continuing illness-related limitations and support psychological well-being and everyday functioning despite the persistence of the disease and its consequences (44). Accordingly, the strong association between AIS and EHP-30 scores observed in the present study may reflect not only the burden of disease itself, but also differences in how women psychologically adapt to and function with the continuing consequences of endometriosis.

The mediation analysis identified a statistically significant indirect association between financial toxicity and HRQoL through disease acceptance. Given the cross-sectional design, this finding should not be interpreted as evidence of a temporal or causal pathway. Rather, the mediation analysis represents a statistical decomposition of concurrently assessed associations, with the direction of the model specified on theoretical grounds. Alternative temporal sequences remain plausible; for example, poorer HRQoL may contribute to reduced occupational functioning and greater financial strain, while greater disease burden may simultaneously affect financial well-being, disease acceptance, and HRQoL. Bargiel-Matusiewicz et al. demonstrated that illness acceptance partially mediated the association between social support and quality of life in women with Hashimoto's disease, providing evidence that illness acceptance may function as a statistical mediator between psychosocial resources and perceived quality of life in chronic illness (48). From the perspective of financial toxicity, Zhao et al. similarly found that loneliness partially mediated the association between financial toxicity and quality of life among older adults with cancer, providing further evidence that psychosocial factors may contribute to the statistical association between financial strain and quality of life (49). In the present study, the persistence of a statistically significant direct association between financial toxicity and HRQoL after accounting for disease acceptance was consistent with partial rather than complete statistical mediation. Disease acceptance may therefore represent one component of a broader set of factors underlying the association between financial toxicity and HRQoL. Longitudinal studies with repeated assessments of these constructs are therefore required to determine their temporal ordering and to test the hypothesised pathway.

Importantly, disease acceptance should be considered within a broader network of psychological processes relevant to adjustment to endometriosis. Recent longitudinal evidence showed that greater pain catastrophizing was associated with subsequent depressive symptoms both directly and indirectly through greater pain interference (25). In another domain, body functionality appreciation statistically mediated the association between dyspareunia severity and sexual distress (26). Although these studies examined different outcomes and did not assess disease acceptance, they indicate that cognitive and adaptive responses to the consequences of endometriosis may be relevant to different dimensions of patients' well-being. Disease acceptance may therefore represent one component of a broader psychosocial adaptation process rather than an isolated mechanism underlying HRQoL.

Such a perspective is particularly relevant given the multidimensional impact of endometriosis across physical and psychological well-being, sexual and intimate relationships, reproductive health, and social and occupational functioning, as highlighted in recent evidence (6) This broader biopsychosocial context is compatible with multidimensional conceptualizations of HRQoL, including the Wilson–Cleary model, which describes quality of life in relation to interconnected biological and physiological factors, symptom status, functional status, general health perceptions, and individual and environmental characteristics (28). Within this framework, pain burden, psychological distress, and coping processes have been associated with HRQoL in women with endometriosis (47, 50). Whether these or other factors contribute to the remaining association between financial toxicity and HRQoL after accounting for disease acceptance was not examined in the present study and warrants investigation in future, preferably longitudinal, research. Disease acceptance may therefore account for part, but not all, of the association between financial toxicity and HRQoL in women with endometriosis, highlighting psychological adaptation as one potential component of this relationship.

The distinction between patient-reported financial toxicity and conventional socioeconomic indicators is consistent with the conceptualization of financial toxicity as a patient-reported construct that cannot be reduced to income or healthcare expenditure alone. In the original validation of the COST measure, de Souza et al. reported only a modest correlation between COST scores and household income, while COST remained associated with quality of life after accounting for income and other sociodemographic and clinical characteristics (16).

A similar pattern was reported by Baddour et al., who examined objective financial metrics alongside patient-reported financial toxicity. Although lower earnings were associated with worse COST scores, out-of-pocket expenditure alone was not significantly associated with subjective financial toxicity; rather, financial toxicity was greater when these expenditures represented a larger proportion of patients' earnings (51). These findings illustrate that the subjective financial impact of illness may depend not only on the absolute level of income or expenditure, but on how financial demands relate to the resources available to the individual. Individuals with comparable household incomes may therefore experience different levels of financial strain depending on their illness-related financial demands and broader individual circumstances. From a patient-centered perspective, this distinction is particularly relevant because conventional socioeconomic indicators describe patients' financial resources but do not fully capture how financially burdensome living with and managing a chronic condition is experienced by the individual. The present findings therefore support the value of incorporating patient-reported financial toxicity alongside conventional socioeconomic indicators when examining HRQoL in women with endometriosis.

Among the covariates included in the model, older age and a history of infertility treatment were associated with lower EHP-30 scores, indicating better HRQoL, whereas previous surgical treatment was not associated with HRQoL. Evidence regarding the relationship between age and HRQoL in women with endometriosis has been inconsistent. While some studies have reported poorer HRQoL with increasing age (20, 52), others have observed better quality of life among older women (53) or a weak inverse association between age and the EHP-30 pain subscale (8). This variability is also reflected in broader evidence reviewed by Breton et al. (54). The finding regarding infertility treatment should be interpreted more cautiously, as previous studies have generally highlighted infertility as a factor associated with poorer HRQoL (53), whereas Pontoppidan et al. found no association between prolonged attempts to conceive and EHP-30 scores (55). Importantly, the present study assessed a history of infertility treatment rather than current infertility status or infertility-related burden. Women who had and had not undergone infertility treatment may also differ in unmeasured clinical, reproductive, healthcare-access, or socioeconomic characteristics. Moreover, infertility treatment was included as a covariate rather than as a primary exposure of interest; therefore, this secondary association should not be interpreted as evidence that infertility treatment itself is associated with improved HRQoL. Finally, the absence of an association with previous surgical treatment adds to mixed findings regarding surgical history and HRQoL (56, 57). Overall, associations involving covariates were secondary to the primary study hypotheses and should be interpreted cautiously.

## 5 Clinical implications

The present findings have potential implications for patient-centered care in endometriosis. The independent associations of patient-reported financial toxicity with both disease acceptance and HRQoL suggest that financial well-being may represent a relevant dimension of the broader patient experience. Importantly, assessment of socioeconomic circumstances based on household income alone may not fully capture the financial strain experienced by individual women. Incorporating patient-reported financial concerns into holistic assessment may therefore help identify patients experiencing financial difficulties that are not apparent from conventional socioeconomic indicators. Given the cross-sectional nature of the present findings, financial strain and disease acceptance should be regarded as potentially relevant targets for further investigation rather than established targets for intervention. Longitudinal and intervention-based studies are needed to determine whether modifying either factor is associated with subsequent improvements in HRQoL.

## 6 Limitations

The cross-sectional design limits the interpretation of temporal and causal relationships between financial toxicity, disease acceptance, and HRQoL. Although the mediation analysis showed a statistically significant indirect association through disease acceptance, the direction of these relationships cannot be established, and reciprocal relationships are also possible. Thus, the specified direction of the mediation model should be regarded as theoretically hypothesised rather than temporally established.

Recruitment through online patient communities, social media channels, and endometriosis-related networks may have introduced selection bias. Women engaged in such communities may differ from the broader population of women with endometriosis in disease burden, healthcare experiences, or willingness to participate in research, and the sample was characterized by a relatively high educational and socioeconomic profile, with over 72% of participants holding a higher education degree and more than 75% being permanently employed. These characteristics may limit the generalizability of the findings, particularly to more socioeconomically diverse populations of women with endometriosis. Endometriosis diagnosis and clinical characteristics were self-reported and not verified against medical records, introducing the possibility of recall and reporting bias.

As this study represents a secondary analysis of an existing dataset, no formal *a priori* sample-size calculation was performed for the present analyses. In addition, 65 influential observations were excluded from the primary analytical sample; however, sensitivity analysis using the full post-quality-control sample (*N* = 476) yielded a largely consistent pattern of findings. Finally, dichotomization of household income per capita may have reduced information on potential differences across the original income categories.

The models included selected sociodemographic and clinical covariates; however, other factors potentially related to HRQoL, such as disease phenotype or stage, psychological distress, comorbidities, and treatment status at the time of survey completion, were not included in the present analysis. Importantly, the survey did not include a direct measure of current subjective pain or symptom severity. Given the central role of pain in the burden of endometriosis and its association with psychological functioning and HRQoL, the absence of a measure of current pain intensity represents a potential source of residual confounding. Recent longitudinal evidence demonstrating relationships among pain catastrophizing, pain interference, and subsequent depressive symptoms further highlights the relevance of pain-related processes to psychological functioning in women with endometriosis (25). Accordingly, unmeasured differences in current pain and symptom burden, as well as treatment-related factors, may have contributed to the observed associations among financial toxicity, disease acceptance, and HRQoL.

The study was conducted in Poland, where healthcare system characteristics and the broader socioeconomic context may shape the experience of financial toxicity. This context should be considered when applying the findings to populations in other countries. Longitudinal research incorporating more detailed clinical, symptomatic, and psychosocial characteristics would help clarify the temporal relationships between financial toxicity, disease acceptance, and HRQoL.

## 7 Conclusion

This study found that higher COST-FACIT scores, indicating better financial well-being and lower financial toxicity, were independently associated with better HRQoL and greater disease acceptance in women with endometriosis, while greater disease acceptance was associated with better HRQoL. The observed indirect association between financial toxicity and HRQoL through disease acceptance is consistent with the possibility that psychological adaptation may represent one factor involved in this relationship, although the cross-sectional design does not allow temporal ordering or causal relationships to be established. Importantly, patient-reported financial toxicity was associated with both disease acceptance and HRQoL independently of household income, highlighting that subjective financial strain captures an aspect of the patient experience that is not reflected by income alone. These findings support the relevance of considering both financial well-being and psychological adaptation within a patient-centered understanding of HRQoL in women with endometriosis.

## Data Availability

The raw data supporting the conclusions of this article will be made available by the authors, without undue reservation.
