# Supplementary material for: Financial toxicity, disease acceptance, and health-related quality of life in women with endometriosis: a cross-sectional mediation analysis
Source: Front Med (Lausanne). 2026 Oct 22;13:1983927. doi: 10.3389/fmed.2026.1983927 (PMC13736637; doi:10.3389/fmed.2026.1983927)
Supplement: Supplementary file 1 [file Table1.docx]

**Table S1.** Full data set regression-based mediation analysis examining the association between patient-reported financial toxicity and health-related quality of life through disease acceptance.

| Antecedent | Consequent | | | | | | | | | | | | | | |  |
| --- | --- | --- | --- | --- | --- | --- | --- | --- | --- | --- | --- | --- | --- | --- | --- | --- |
|  | Acceptance of illness [AIS] | | | | | |  | | Quality of life [EHP-30] | | | | | | |  |
|  | *B* | *SE* | *95% CI* | | *t* | *p* | |  | | *B* | *SE* | *95% CI* | | *t* | *p* | |
|  |  |  | *LL* | *UL* |  |  |  |  | |  |  | *LL* | *UL* |  |  |  |
| Financial Toxicity [COST-FACIT] | 0.554 | 0.035 | 0.483 | 0.626 | 15.702 | <0.001 | |  | | -0.611 | 0.088 | -0.810 | -0.401 | -6.974 | <0.001 | |
| Acceptance of illness [AIS] | –– | –– | –– | –– | –– | –– | |  | | -0.960 | 0.093 | -1.169 | -0.751 | -10.352 | <0.001 | |
| Income  ≤5 000 → >5 000 | 0.328 | 0.676 | -1.014 | 1.652 | 0.486 | 0.627 | |  | | 0.511 | 1.359 | -2.139 | 3.234 | 0.376 | 0.707 | |
| Age | 0.116 | 0.046 | 0.024 | 0.206 | 2.492 | 0.013 | |  | | -0.258 | 0.094 | -0.431 | -0.084 | -2.747 | 0.006 | |
| Surgical treatment  No → Yes | -1.232 | 0.686 | -2.593 | 0.111 | -1.796 | 0.073 | |  | | -0.563 | 1.383 | -3.432 | 2.249 | -0.407 | 0.684 | |
| Infertility treatment  No → Yes | -0.256 | 0.739 | -1.736 | 1.209 | -0.347 | 0.729 | |  | | -1.588 | 1.484 | -4.677 | 1.603 | -1.070 | 0.285 | |
| Constant | 22.525 | 0.359 | 21.796 | 23.262 | 62.812 | <0.001 | |  | | 62.185 | 0.721 | 60.755 | 63.665 | 86.280 | <0.001 | |
| Model fit | $R^{2}= 0.398,$ $R_{\mathrm{adj}}^{2}= 0.391,$ *F*_(5, 470)_ = 62.080, *p* < 0.001 | | | | | |  | | $R^{2}= 0.483,$ $R_{\mathrm{adj}}^{2}= 0.476,$  *F*_(6, 469)_ = 72.917, *p* < 0.001 | | | | | | |  |

**Note:** AIS – Acceptance of Illness Scale; COST-FACIT – Comprehensive Score for Financial Toxicity; EHP-30– Endometriosis Health Profile; *B* – unstandardized regression coefficient; *SE* – bootstrap standard error; *CI* – bootstrap confidence interval; *LL* – lower limit; *UL* – upper limit; *t* – *t*-statistic; *p* – *p*-value; *R*² – coefficient of determination; adjusted *R*² – adjusted coefficient of determination; *F* – *F*-statistic. Standard errors and 95% confidence intervals were estimated using 5,000 bootstrap samples.
