# Supplementary material for: Financial toxicity, disease acceptance, and health-related quality of life in women with endometriosis: a cross-sectional mediation analysis
Source: Front Med (Lausanne). 2026 Oct 22;13:1983927. doi: 10.3389/fmed.2026.1983927 (PMC13736637; doi:10.3389/fmed.2026.1983927)
Supplement: Supplementary file 2 [file Table2.docx]

| Section | Item | Recommendation | Page / location in manuscript |
| --- | --- | --- | --- |
| Title and abstract | 1(a) | Indicate the study's design with a commonly used term in the title or the abstract | p. 1 |
|  | 1(b) | Provide in the abstract an informative and balanced summary of what was done and what was found | p. 1 |
| Introduction | 2 | Explain the scientific background and rationale for the investigation being reported | pp. 2-4 |
|  | 3 | State specific objectives, including any prespecified hypotheses | p. 4 |
| Methods | 4 | Present key elements of study design early in the paper | p. 4 |
|  | 5 | Describe the setting, locations, and relevant dates, including periods of recruitment, exposure, follow-up, and data collection | pp. 4-5 |
|  | 6(a) | Give the eligibility criteria, and the sources and methods of selection of participants | p. 5 |
|  | 7 | Clearly define all outcomes, exposures, predictors, potential confounders, and effect modifiers. Give diagnostic criteria, if applicable | pp. 7-9 |
|  | 8* | For each variable of interest, give sources of data and details of methods of assessment (measurement). Describe comparability of assessment methods if there is more than one group | pp. 7-8 |
|  | 9 | Describe any efforts to address potential sources of bias | pp. 8 |
|  | 10 | Explain how the study size was arrived at | pp. 5-6 |
|  | 11 | Explain how quantitative variables were handled in the analyses. If applicable, describe which groupings were chosen and why | p. 9 |
|  | 12(a) | Describe all statistical methods, including those used to control for confounding | p. 8-9 |
|  | 12(b) | Describe any methods used to examine subgroups and interactions | N/A - no subgroup or interaction analyses |
|  | 12(c) | Explain how missing data were addressed | p. 9 |
|  | 12(d) | If applicable, describe analytical methods taking account of sampling strategy | N/A - no design-based sampling analysis |
|  | 12(e) | Describe any sensitivity analyses | p. 6; Supplementary Table S1 |
| Results | 13(a) | Report numbers of individuals at each stage of study | pp. 5-6; Figure 1 |
|  | 13(b) | Give reasons for non-participation at each stage | pp. 5-6; Figure 1 |
|  | 13(c) | Consider use of a flow diagram | p. 6; Figure 1 |
|  | 14(a) | Give characteristics of study participants and information on exposures and potential confounders | pp. 10-12; Table 1 |
|  | 14(b) | Indicate number of participants with missing data for each variable of interest | p. 10 |
|  | 15* | Report numbers of outcome events or summary measures | Not reported in the current version |
|  | 16(a) | Give unadjusted estimates and, if applicable, confounder-adjusted estimates and their precision. Make clear which confounders were adjusted for and why they were included | pp. 9, 11-13; Table 2 - adjusted estimates and 95% CIs reported |
|  | 16(b) | Report category boundaries when continuous variables were categorized | pp. 9-12; Table 1 |
|  | 16(c) | If relevant, consider translating estimates of relative risk into absolute risk for a meaningful time period | N/A - no relative risk estimates |
|  | 17 | Report other analyses done - eg analyses of subgroups and interactions, and sensitivity analyses | p. 6; Supplementary Table S1 |
| Discussion | 18 | Summarise key results with reference to study objectives | p. 13 |
|  | 19 | Discuss limitations of the study, taking into account sources of potential bias or imprecision. Discuss both direction and magnitude of any potential bias | pp. 18-19 |
|  | 20 | Give a cautious overall interpretation of results considering objectives, limitations, multiplicity of analyses, results from similar studies, and other relevant evidence | pp. 13-19 |
|  | 21 | Discuss the generalisability (external validity) of the study results | pp. 18-19 |
| Other information | 22 | Give the source of funding and the role of the funders for the present study and, if applicable, for the original study on which the present article is based | p. 20 |

**Table S2**. STROBE Statement - Checklist of items that should be included in reports of cross-sectional studies
