# Supplementary material for: Financial toxicity, disease acceptance, and health-related quality of life in women with endometriosis: a cross-sectional mediation analysis
Source: Front Med (Lausanne). 2026 Oct 22;13:1983927. doi: 10.3389/fmed.2026.1983927 (PMC13736637; doi:10.3389/fmed.2026.1983927)
Supplement: Supplementary file 3 [file Table3.docx]

**Table S3.** Diagnostic assessment of assumptions for the regression-based mediation models.

| Model tested | Residuals normality  Kolmogorov-Smirnov | | Homoscedasticity  Breusch-Pagan | | Multicollinearity  Variance Inflation Factor | |
| --- | --- | --- | --- | --- | --- | --- |
|  | *K-S* | *p* | *B-P* | *p* | Antecedent | *VIF* |
| Consequent:  Acceptance of illness [AIS] | 0.037 | 0.615 | 7.424 | 0.191 | Financial Toxicity [COST-FACIT] | 1.23 |
|  |  |  |  |  | Income | 1.22 |
|  |  |  |  |  | Age | 1.21 |
|  |  |  |  |  | Surgical treatment | 1.28 |
|  |  |  |  |  | Infertility treatment | 1.13 |
| Consequent:  Quality of life [EHP-30] | 0.031 | 0.832 | 12.099 | 0.060 | Financial Toxicity [COST-FACIT] | 2.135 |
|  |  |  |  |  | Acceptance of illness [AIS] | 2.001 |
|  |  |  |  |  | Income | 1.22 |
|  |  |  |  |  | Age | 1.22 |
|  |  |  |  |  | Surgical treatment | 1.29 |
|  |  |  |  |  | Infertility treatment | 1.13 |
